# Efficacy of fluralaner chewable tablets (Bravecto^®^) against Asian longhorned tick (*Haemaphysalis* *longicornis*) infestations of dogs

**DOI:** 10.1186/s13071-023-05664-w

**Published:** 2023-02-08

**Authors:** Melissa Petersen, Riaan Maree, Alta Viljoen, Julian E. Liebenberg, Frank Guerino

**Affiliations:** 1 Merck Animal Health, De Soto, KS 66018 USA; 2 Clinvet USA, Waverly, NY 14892 USA; 3 Clinvet South Africa, Bloemfontein, 9338 South Africa; 4 Merck Animal Health, Madison, NJ 07940 USA

**Keywords:** Asian longhorned tick, Bravecto^®^, Dog, Efficacy, Fluralaner chewable, *Haemaphysalislongicornis*

## Abstract

**Background:**

The parthenogenic reproductive ability of *Haemaphysalis* *longicornis*, facilitating quick life cycle completion and rapid geographic spread and its pathogen vector potential make infestations a risk to human and canine health. Two 90-day studies were initiated to evaluate the efficacy of a single fluralaner administration for the treatment and prevention of *H.* *longicornis* infestations on dogs.

**Methods:**

Dogs were randomly assigned (10 dogs/group) to either an untreated control group or a group treated once (Day 0) with 13.64% w/w fluralaner chewable tablets (Bravecto^®^) at the minimum label dose rate of 25 mg/kg. Each dog was infested with approximately 50 *H.* *longicornis* ticks on Days -9 or -6 and on Days -2, 28, 58 and 88. A different US tick isolate was used in each study. Tick counts were completed on Days -7 or -4, 2, 30, 60 and 90. The primary efficacy criterion was a 90% reduction in arithmetic mean tick counts between the treated and control groups. For between-group comparisons at any assessment, at least six control dogs were required to retain at least 25% of the infestation dose (13 live ticks).

**Results:**

Pre-study infestations demonstrated susceptibility of all study dogs to challenge with *H.* *longicornis*. At each subsequent assessment in both studies, at least seven untreated control dogs retained ≥ 25% of the challenge, demonstrating adequate infestations for each efficacy calculation. On Days 2, 30, 60 and 90 the mean live tick infestation rate (number of ticks recovered from each dog/infesting challenge of each dog) of untreated control dogs ranged from 27.8 to 60.8%. No live ticks, free or attached, were found on any fluralaner-treated dog in either study. Between-group differences were statistically significant (*P* ≤ 0.0002) at each assessment.

**Conclusion:**

At the minimum recommended label dose rate of 25 mg/kg, fluralaner chewable tablets were 100% effective in eliminating *H.* *longicornis* ticks from dogs infested at the time of treatment. Complete efficacy against both US isolates of this tick was maintained through 90 days following a single treatment. Therefore, fluralaner is a treatment of choice for protecting dogs against this invasive tick species.

**Graphical abstract:**

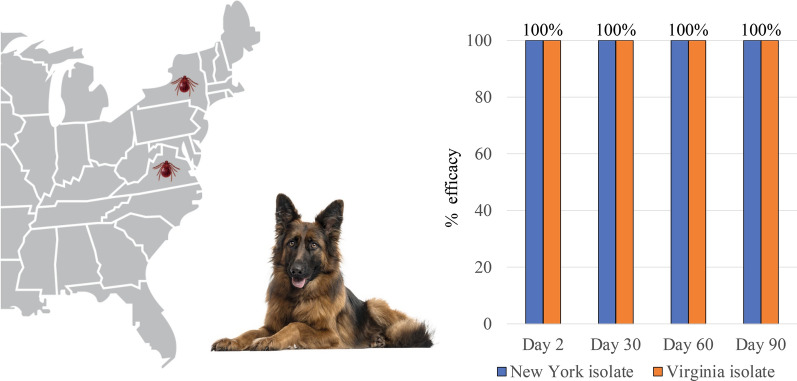

## Background

The Asian longhorned tick, *Haemaphysalis* *longicornis*, is an invasive species with pathogen vector potential, now spreading within the USA and already established as a threat to human and animal health in Australia and New Zealand [1, 2]. Since the first US confirmation on a sheep in New Jersey, infestations have been reported in over 172 counties across 17 states, ranging from New York and Connecticut in the northeast to Arkansas and Missouri in the Midwest and Georgia and the Carolinas in the south [3–5]. Since that New Jersey finding, the USA recording of *H.* *longicornis* infestations in over 25 animal species, including dogs, cats, birds and humans, demonstrates the tick’s broad host range and widening geographic presence [4]. The suitability of *H.* *longicornis* to temperate climates, its parthenogenic and multi-host capability and an ability to overwinter indicate that its spread in the USA will continue [2]. Modeling of its potential expansion predicts that within North America, geographic expansion of the range of *H.* *longicornis* could extend from the Gulf Coast and southeastern USA to Quebec and Nova Scotia in the east and from California to the coast of British Columbia in the west [6–8]. Pathogens reported to be carried by *H.* *longicornis* include thrombocytopenia syndrome virus, *Rickettsia* *japonica*, *Anaplasma*, *Theileria* and *Babesia* species and possibly *Rickettsia* *rickettsiae*, the agent of Rocky Mountain Spotted Fever [9, 10]. That vector potential, the parthenogenic reproduction providing rapid life cycle completion and the rapid spread of the tick’s geographic distribution make infestations a risk to human and animal health and emphasize the need for effective acaricides [5].

When administered orally to dogs at the minimum label dose rate of 25 mg/kg, the acaricidal isoxazoline compound fluralaner has been shown to provide 8-week efficacy against *Amblyomma* *americanum* and 12-week or longer efficacy against *Ixodes* *scapularis*, *Ixodes* *ricinus*, *Ixodes* *holocyclus*, *Rhipicephalus* *sanguineus*, *Dermacentor* *reticulatus* and fleas as well as effectiveness against demodectic and sarcoptic mites [11–15]. A dose-ranging study conducted with a Japanese strain of *H.* *longicornis* (Okayama) indicated that that the minimum label dose rate would provide high and long-lasting efficacy against this tick for 114 days [16]. To confirm the results of that study and to establish a fluralaner claim for efficacy, laboratory studies were initiated against two different US isolates of *H.* *longicornis*.

## Methods

Two separate 90-day studies were conducted, at two different study sites, with two different US tick isolates to evaluate the effectiveness of a single fluralaner administration for the treatment and prevention of *H.* *longicornis* infestations on dogs. The in-life phase of Study 1 was conducted during February to May and the in-life phase of Study 2 was conducted from July to October. Each study was a completely randomized design with a fluralaner-treated group and an untreated control group. Treatment was administered by unmasked individuals. All observations and procedures, including general health observations, tick infestations and tick counts, were performed by masked individuals. Both studies were conducted in accordance with Good Clinical Practices and the World Association for the Advancement of Veterinary Parasitology (WAAVP) guidelines for evaluating the efficacy of parasiticides against flea and tick infestations of dogs and cats [17, 18].

### Animals

Prior to beginning each study, 24 dogs in Study 1 and 30 dogs in Study 2 were acclimated to kennel conditions beginning on Day 10. To be enrolled in either study, dogs were required to be clinically healthy with no pre-existing conditions (e.g., injury, trauma, disease) that could have affected the outcome, at least 6 months of age, not treated with any long-acting anti-flea or anti-tick product within the previous 180 days and have demonstrated susceptibility to tick infestation based on retaining at least 13 live ticks or ≥ 25% of a pre-study infestation. In that pre-study period and then throughout the study, dogs were housed in individual cages (except for removal for tick infestations) in a thermostatically controlled environment with an approximate 12-h light/12-h dark cycle, fed a commercial dog food per site practice and allowed ad libitum access to water. Cages were segregated by treatment group to avoid cross exposure of dogs to fluralaner. From the dogs presented for acclimation in each study, 20 were retained for experimental purposes. Dogs selected for Study 1 were Beagles, 11 males and 9 females, aged from 2 to 4 years and weighing from 10.2 to 14.6 kg. Study 2 included 13 Beagles and 7 mixed breed dogs, 7 males and 13 females, aged from 2 to 7 years and weighing from 10.2 to 13.3 kg.

### Randomization and treatment

On Day 3, dogs were randomized to treatment or control groups in a 1:1 ratio using a computer-generated randomization table such that 10 dogs were included in each group. Dogs in Group 1 remained untreated but were handled in the same manner as Group 2 dogs to have a reference time for scheduling post-treatment activities. Group 2 dogs received a single treatment, on Day 0, with 13.64% w/w fluralaner chewable tablets (Bravecto^®^, Merck Animal Health, Madison, NJ, USA) based on body weights recorded on Day 2. One or more whole chewable fluralaner tablets were administered, at doses as close as possible to the minimum recommended dose of 25 mg/kg without underdosing. Actual doses ranged from 25.2 to 35.9 mg/kg. Treatment was administered within 20 min after feed had been offered. Immediately after treatment, dogs were returned to their cages and observed for approximately 1 h for adverse events and to ensure the treatment was retained. Additionally, 3-h and 6-h post-treatment observations were made. Post-treatment observations on all dogs were conducted in a random order of evaluation.

### Tick infestations and counts

At the pre-study infestations on Day -6 (Study 1) or -9 (Study 2) and on Day -2, 28, 58 and 88, the 20 dogs included in each study were infested with approximately 50 adult, unfed female *H.* *longicornis* ticks. The *H.* *longicornis* isolate for Study 1 had been collected in New York in July 2018, and for Study 2 the isolate was obtained from vegetation in Virginia in October 2018. The ticks were identified as *H.* *longicornis* based on morphology. The tick isolates were reared on laboratory rabbits with no tick-borne infections. Ticks were stored in individually labeled vials with the tick species, isolate and date of collection. These vials were stored in dedicated containers, which maintained appropriate humidity conditions. Prior to the infestation, each dog was sedated (dexmedetomidine hydrochloride, 0.5 mg/ml Dexdomitor^®^, Zoetis), and a vial containing the ticks was emptied onto the dog’s back. Each dog was then held individually in an infestation chamber (dimensions: Study 1, 0.9 × 1.1 × 0.9 m; Study 2, 0.8 × 0.9 × 0.7 m) for a period of up to approximately 4 h to ensure that ticks had established. After the infestation period, the dog was removed from the infestation chamber and returned to its cage. To avoid any cross-animal exposure to fluralaner, from Day 0 forward an infestation chamber was assigned to each dog and used only for that dog for the duration of the study. Before each use, infestation chambers were thoroughly washed with soap and water, rinsed with clean water and double rinsed with isopropyl alcohol (with air drying between each alcohol rinse).

On each count day, at approximately 48 h after infestation, each dog was examined for ticks by pushing the hair against its natural nap to expose the skin, covering the entire dog’s body. Any ticks found were gently removed using either forceps or fingers. After this examination was complete, the dog was combed (tick comb, approximately 32 teeth per 2.5 cm) to recover any ticks that could have been missed during the visual inspection. Collected live ticks were classified as either free (on the host) or attached and then discarded. The tick collection procedure on each dog lasted a minimum of 5 min.

### Number of dogs

According to the World Association for the Advancement of Veterinary Parasitology (WAAVP) guideline for studies investigating the efficacy of ectoparasiticides, a minimum of six dogs is recommended for each treatment group [18]. A sample size of 10 dogs per treatment group was used in this study to help ensure that an adequate infestation (≥ 13 ticks) was achieved in a minimum of six untreated control dogs.

### Statistical analysis

The primary endpoint was based on between-group differences in live tick counts, attached and free on the host, with the experimental unit the individual dog. At least six dogs in the control group were required to be adequately infested (a minimum of 13 live ticks or 25% of the infestation dose) to allow comparisons at any time point. The mean live tick infestation rate was calculated as the number of ticks recovered from each dog/infestation challenge for each dog. Tick counts were analyzed using a linear mixed model that included treatment group as a fixed effect. The fluralaner group and the control group were compared using a two-sided test at a 5% level of significance. Separate analyses were conducted at each tick infestation day. Arithmetic least squares means were used for treatment comparisons. The null hypothesis was that there was no significant difference between the treated group and the control group. The primary software used for analysis was SAS version 9.3 or higher.

Within each infestation schedule, the primary efficacy endpoint was calculated using the formula below, based on arithmetic mean tick counts:

$${\text{Efficacy }}\left( \% \right) \, = \, \left[ {\left( {{\text{Group 1 mean counts }}-{\text{ Group 2 mean counts}}} \right)/{\text{ Group 1 mean counts}}} \right] \, \times 100$$

## Results and discussion

The initial infestations (Day -9 or -6) demonstrated the susceptibility of all study dogs to the laboratory infestations, in Study 1 ranging from 13 to 36 live attached ticks and in Study 2 from 13 to 34 live attached ticks. After inclusion in Study 2, an untreated control group dog was removed (on Day 22) following a seizure. There were no treatment-related adverse events.

At each time point in both studies, at least seven dogs in Group 1 (untreated controls) had ≥ 25% of the *H.* *longicornis* challenge, demonstrating an adequate tick infestation at each assessment to provide valid between-group comparisons. On Days 2, 30, 60 and 90, the mean live tick infestation rate (number of ticks recovered from each dog/infesting challenge for each dog) of dogs in the negative control group ranged from a low of 27.8% (Study 1) to a high of 60.8% (Study 2) (Table 1).

**Table 1:** *Haemaphysalis* *longicornis* infestation rate in control dogs

Day of count	Number (%) of control group dogs with adequate infestations		Mean infestation rate (%)^a^	
	Study 1	Study 2^b^	Study 1	Study 2
2	9/10 (90.0)	10/10 (100.0)	40.4	60.8
30	8/10 (80.0)	7/9 (77.8)	36.8	40.2
60	9/10 (90.0)	8/9 (88.9)	32.2	40.9
90	7/10 (70.0)	8/9 (88.9)	27.8	37.6

^a^Infestation rate calculated as: (number of live ticks recovered from each dog/infesting challenge for each dog) × 100

^b^Ten dogs on Day 2; one untreated control dog was removed on Day 22 following a seizure

No live ticks, free or attached, were found on any fluralaner-treated dog in either study. At each post-treatment assessment, the differences in live tick counts between the fluralaner-treated and the control group were statistically significant (*P* ≤ 0.0002) (Tables 2, 3). Fluralaner treatment resulted in a 100% reduction against existing tick infestations at 48 h post administration, and 100% efficacy against *H.* *longicornis* infestation was maintained for the full 90-day study period. The fluralaner formulation tested in these studies and a topical fluralaner formulation are the only isoxazoline products labeled to provide up to 12 weeks of acaricidal activity following a single treatment [19, 20]. The finding of 100% efficacy of this oral formulation against both tested strains of *H.* *longicornis* in these two studies confirms the earlier dose-ranging study and shows that, as against other tick species, fluralaner remains highly effective for at least 12 weeks. That high fluralaner efficacy against both the recent US isolates, as well as against the Japanese strain tested in an earlier study [16], compares favorably with other investigations of isoxazolines, all requiring monthly retreatments, against *H.* *longicornis*.

**Table 2:** Study 1, live *Haemaphysalis* *longicornis* tick counts

	Untreated control dogs (n = 10)			Fluralaner-treated dogs (n = 10)			% efficacy	Test statistic^a^	
	Attached	Free	Total	Attached	Free	Total	Fluralaner vs. control		
Day 2 counts									
Arithmetic mean	15.8	4.4	20.2	0.0	0.0	0.0	100.0	*t*_18_ = 9.38	*P* < 0.0001
Standard deviation	10.7	9.3	6.8	0.0	0.0	0.0			
Median	18.0	0.0	21.0	0.0	0.0	0.0			
Range	0–30	0–23	6–30	0–0	0–0	0–0			
Day 30 counts									
Arithmetic mean	18.4	0.0	18.4	0.0	0.0	0.0	100.0	*t*_18_ = 8.77	*P* < 0.0001
Standard deviation	6.6	0.0	6.6	0.0	0.0	0.0			
Median	18.0	0.0	18.0	0.0	0.0	0.0			
Range	10–33	0–0	10–33	0–0	0–0	0–0			
Day 60 counts									
Arithmetic mean	16.1	0.0	16.1	0.0	0.0	0.0	100.0	*t*_18_ = 11.75	*P* < 0.0001
Standard deviation	4.3	0.0	4.3	0.0	0.0	0.0			
Median	17.0	0.0	17.0	0.0	0.0	0.0			
Range	5–21	0–0	5–21	0–0	0–0	0–0			
Day 90 counts									
Arithmetic mean	13.9	0.0	13.9	0.0	0.0	0.0	100.0	*t*_18_ = 4.76	*P* = 0.0002
Standard deviation	9.2	0.0	9.2	0.0	0.0	0.0			
Median	17.5	0.0	17.5	0.0	0.0	0.0			
Range	0–24	0–0	0–24	0–0	0–0	0–0			

^a^Based on 18 degrees of freedom (*df*) in the denominator for the analysis done for each count day

**Table 3:** Study 2, live *Haemaphysalis* *longicornis* tick counts

	Untreated control dogs (*n* = 9)^a^			Fluralaner-treated dogs (*n* = 10)			% efficacy	Test statistic^b^	
	Attached	Free	Total	Attached	Free	Total	Fluralaner vs. control		
Day 2 counts									
Arithmetic mean	30.2	0.2	30.4	0.0	0.0	0.0	100.0	*t*_18_ = 14.99	*P* < 0.0001
Standard deviation	6.6	0.4	6.4	0.0	0.0	0.0			
Median	30.0	0.0	30.5	0.0	0.0	0.0			
Range	19–38	0–1	20–38	0–0	0–0	0–0			
Day 30 counts									
Arithmetic mean	20.1	0.0	20.1	0.0	0.0	0.0	100.0	*t*_17_ = 5.09	*P* < 0.0001
Standard deviation	12.5	0.0	12.5	0.0	0.0	0.0			
Median	18.0	0.0	18.0	0.0	0.0	0.0			
Range	1–40	0–0	1–40	0–0	0–0	0–0			
Day 60 counts									
Arithmetic mean	20.3	0.1	20.4	0.0	0.0	0.0	100.0	*t*_17_ = 6.79	*P* < 0.0001
Standard deviation	9.4	0.3	9.6	0.0	0.0	0.0			
Median	22.0	0.0	22.0	0.0	0.0	0.0			
Range	1–33	0–1	1–33	0–0	0–0	0–0			
Day 90 counts									
Arithmetic mean	18.7	0.1	18.8	0.0	0.0	0.0	100.0	*t*_17_ = 6.75	*P* < 0.0001
Standard deviation	8.7	0.3	8.8	0.0	0.0	0.0			
Median	18.0	0.0	18.0	0.0	0.0	0.0			
Range	1–30	0–1	1–30	0–0	0–0	0–0			

^a^Ten dogs on Day 2; one untreated control dog was removed on Day 22 following a seizure

^b^Based on 18 degrees of freedom (*df*) in the denominator for the analysis of the Day 2 counts and 17 *df* in the denominator for each analysis of the subsequent count days

The single study reported with afoxolaner indicated that its continuing efficacy against *H.* *longicornis* may not be as high as that of other isoxazolines [21]. While at 48-h post treatment efficacy against infestations present at the time of administration was 100%, against subsequent infestations with the Japanese isolate efficacy had declined to 97.7% at 14 days post treatment and 91.9% at 28 days post-treatment. In that study, calculations were based on geometric means which typically provide a higher efficacy number than do those based on arithmetic mean tick counts. For a single-entity sarolaner formulation, arithmetic mean efficacy against the nymphal stage of a Japanese *H.* *longicornis* isolate was 100% through 21 days post-treatment, 98.7% at 28 days post-treatment when two of seven treated dogs (28.5%) were positive and 95.9% at 35 days post-treatment when three of seven dogs were positive for live ticks [22]. In another study, 48-h post-lotilaner treatment of dogs infested with a Japanese isolate, the arithmetic mean reduction in longhorned tick numbers was 96.8%, with five of the eight study dogs remaining infested with a single tick. High but incomplete efficacy was then maintained at subsequent weekly infestations placed through 35 days following treatment (94.7–99.5%), and up to three dogs were infested with one or two live ticks at each assessment. [23].

Vigilance and effective tick control measures are important in limiting the expansion of *H.* *longicornis* and in maintaining canine health. While no product can reasonably be expected to provide 100% efficacy on 100% of occasions, the consistent and extended high efficacy of fluralaner shown in these studies, including against two separate and recent US isolates, makes it a valuable option for the treatment and control of *H.* *longicornis* infestations on dogs. For this tick species in particular, such high efficacy is important as its parthenogenic capability means that a single female tick can produce an entire population in a new location [24].

## Conclusion

Fluralaner chewable tablets were 100% effective in eliminating adult *H.* *longicornis* ticks from dogs infested at the time of treatment. Complete efficacy was maintained throughout at least 12 weeks following treatment against two *H.* *longicornis* strains isolated in the USA. Fluralaner is a treatment of choice for protecting dogs against this invasive tick species.

## Data Availability

Data from this study are proprietary and maintained by Merck Animal Health, Madison, NJ, USA.
